# Oxaliplatin-Fluoropyrimidine Combination (XELOX) Therapy Does Not Affect Plasma Amino Acid Levels and Plasma Markers of Oxidative Stress in Colorectal Cancer Surgery Patients: A Pilot Study

**DOI:** 10.3390/nu11112667

**Published:** 2019-11-05

**Authors:** Roberto Aquilani, Silvia Brugnatelli, Maurizia Dossena, Roberto Maestri, Sara Delfanti, Daniela Buonocore, Federica Boschi, Elena Simeti, Anna Maria Condino, Manuela Verri

**Affiliations:** 1 Department of Biology and Biotechnology “Lazzaro Spallanzani”, University of Pavia, 27100 Pavia, Italy; dottore.aquilani@gmail.com (R.A.); maurizia.dossena@unipv.it (M.D.); daniela.buonocore@unipv.it (D.B.); elena.simeti01@universitadipavia.it (E.S.); 2 Medical Oncology Division, Fondazione IRCCS, Policlinico San Matteo, 27100 Pavia, Italy; S.Brugnatelli@smatteo.pv.it (S.B.); S.DelFanti@smatteo.pv.it (S.D.); 3 Department of Biomedical Engineering of the Montescano Institute, Istituti Clinici Scientifici Maugeri IRCCS, 27040 Montescano (PV), Italy; roberto.maestri@icsmaugeri.it; 4 Department of Drug Sciences, University of Pavia, 27100 Pavia, Italy; federica.boschi@unipv.it (F.B.); annamaria.condino@unipv.it (A.M.C.)

**Keywords:** colorectal cancer, surgery, chemotherapy, plasma amino acids, oxidative stress

## Abstract

Chemotherapy for colorectal cancer may lower muscle protein synthesis and increase oxidative stress. We hypothesize that chemotherapy may worsen plasma amino acids (AAs) and markers of oxidative stress (MOS). Therefore, this study aimed to document plasma AAs and MOS before, during and after chemotherapy in colorectal cancer (CRC) surgery patients. Fourteen normal-weight CRC patients were enrolled one month after surgery and scheduled for oxaliplatin-fluoropyrimidine combination (XELOX) therapy. Venous blood samples for AA and MOS (malondialdehyde, MDA; 8-hydroxy-2’-deoxyguanosine, 8-OHdG) measurements were drawn in fasting patients before each oxaliplatin infusion at initiation (A), 1 month (B) and 3 months (C) of the therapy, and after XELOX had finished (6 months, D). The results showed that during XELOX therapy (from phase B to phase D), in comparison to baseline (phase A), the branched chain amino acid/essential amino acid ratio, branched chain amino acids expressed as a percentage of total AAs, and arginine expressed as a percentage of total AAs significantly decreased (*p* = 0.017, *p* = 0.028, *p* = 0.028, respectively). Plasma levels of MOS did not change significantly. This study indicates that XELOX therapy does not affect plasma AA levels or worsen oxidative stress.

## 1. Introduction

Colorectal cancer (CRC) is the third most common cancer worldwide, the fourth cause of cancer-related deaths [1], and is expected to increase by 60% by 2030 [2]. Even after radical resection, CRC recurrence is still a major concern [3,4]. The treatments for CRC include surgery, chemotherapy and/or radiotherapy, which are invasive treatments often associated with adverse side effects. Recently, immune checkpoint molecules such as programmed cell death-1 (PD-1) and PD ligand-1 (PD-L1) have been identified as a possible target for immunotherapy in microsatellite instability (MSI) tumors among CRC subtypes [5]. In addition, new therapeutic approaches in cancer consider DNA damage and repair inhibitors, which have positively impacted disease outcomes. Despite these therapeutic approaches, the prognosis remains poor for most CRCs, especially when relapsed.

These factors, along with the fact that the progression of CRC is generally slow and patients may be asymptomatic in the early stages of the disease, led researchers to investigate new biomarkers that may be more sensitive than classic CA 19-9 and CEA markers to improve diagnosis and monitor recurrences. Emerging studies suggest that plasma amino acid (AA) measures may be used as good biomarkers: not only do plasma amino acid levels (PAL) vary greatly between cancer patients and healthy subjects [6,7,8,9,10,11], but they may also vary among cancer types [12].

Several studies carried out on CRC patients reported low plasma AA concentrations even in the early stages of the disease [9,12,13,14,15]. All these studies reported low threonine levels, 80% of them reported low leucine, valine and tyrosine, 40% of them reported low lysine, phenylalanine, isoleucine, tryptophan, histidine, glycine and citrulline and 20% of them reported low alanine, aspartate, glutamine, ornithine and arginine. In summary, low plasma levels of one or more essential amino acids were found in 40%–100% of the studies on CRC patients.

To the best of our knowledge, so far no studies have investigated PAL and markers of oxidative stress (MOS) before, during and at the end of a cycle of chemotherapy with combined oxaliplatin + fluoropyrimidine drugs (XELOX) in ambulatory CRC surgery patients.

XELOX is standard treatment for resected CRC patients with a high risk of recurrence. In the present study, we formulated the hypothesis that XELOX therapy may impair post-surgery PAL and increase MOS. This hypothesis was based on the following considerations. Firstly, some studies reported oxaliplatin-induced reductions in muscle protein synthesis and protein content, muscle atrophy and muscle loss [16,17]. Following our reasoning, this could lead to a prevalence of muscle catabolic processes with consequent increased amino acid efflux into the blood stream. Secondly, oxaliplatin increases the production of mitochondrial reactive oxygen species (ROS) that are responsible for increased oxidative stress [16]. XELOX-induced PAL alterations and MOS may cause skeletal muscle atrophy, weakness and fatigue [18,19] and may amplify possible patient malnutrition because of XELOX-induced loss of appetite, enteric neuropathy and gastrointestinal dysfunction [20,21].

Therefore, in the current, prospective, observational, longitudinal cohort pilot study we measured PAL and MOS in CRC surgery patients before, during and at the end of the XELOX cycle, and correlated circulating AAs with circulating MOS, due to the fact that AAs are essential intermediates of tricarboxylic acid (TCA) cycle in the mitochondria, the main site of free radical species formation.

## 2. Materials and Methods

### 2.1. Population

Fourteen ambulatory patients who had been scheduled to receive oxaliplatin-based chemotherapy associated with fluoropyrimidine (= capecitabine) (XELOX regimen) were enrolled in this study.

Oxaliplatin is a widely used type of chemotherapy, mostly for gastrointestinal tumors, among which colorectal cancer (CRC). Oxaliplatin-based chemotherapy has been successfully used for the management of advanced CRC and is currently the regimen of choice for adjuvant treatment for patients with resected colorectal cancer with a high risk of relapse (B3 with high risk features, C1 and C2 Duke’s stages).

The patients had undergone surgery for radical CRC resection (*n* = 7 with open surgery; *n* = 7 with videolaparoscopic surgery). At histological examinations adenocarcinoma types were moderately differentiated (G2) and badly differentiated (G3) (Table 1).

The first cycle of chemotherapy was started about 21 days after discharge from the surgical environment. Each cycle of XELOX therapy consisted in oxaliplatin infusion on Day l (130 mg/m^2^) through a peripheral vein or central venous catheter (CVC), followed by 14 days of oral capecitabine intake (1000 mg/m^2^ per os bis in day), followed, in turn, by 7 days of capecitabine washout. In total, eight cycles of XELOX therapy were scheduled over about 6 months.

Plasma AAs and MOS were measured in venous blood drawn from the antecubital vein in fasting patients between 8 and 9 am at the following phases: before the first cycle of XELOX (phase A), at 1 month (phase B) and at 3 months of the treatment (phase C). During phases A, B and C, blood samples were drawn before the infusion of oxaliplatin. During the 6-month infusion (phase D), blood was drawn at the end of the 21-day cycle.

Table 2 describes the demographic and anthropometric characteristics, and the biohumoral variables that were adopted in our department to monitor the main negative impacts of chemotherapy in no target organs. 

The study was approved by the Ethical-Scientific Committee of Policlinico S. Matteo (Pavia). The patients gave written consent after being well informed about the study characteristics. 

### 2.2. Procedures

Blood samples were delivered to the laboratory where plasma was obtained from heparinized blood using centrifugation (800× *g*, 15 min).

In addition to routine biohumoral parameters, amino acid and oxidative stress marker concentrations were assessed.

#### 2.2.1. Amino Acids

The concentration of free amino acids in the plasma was measured using an AminoQuant II amino acid analyzer, based on the HP 1090 HPLC system, with fully automated precolumn derivatization. Both orthopthalaldehyde (OPA) and 9-fluorenyl-methyl-chloroformate (FMOC) reaction chemistries were used, according to the manufacturer’s protocol. Measurements were made by injecting 1 μL of the derivatized mixture and measuring absorbance simultaneously at 338 and 262 nm [23]. Plasma concentrations were expressed as μmol/L. The measurements of the plasma amino acids were carried out in triplicate by the same laboratory. The mean of the three measurements was calculated and adopted. The characteristics of the method were based both on precision and standardization properties (unpublished data): precision, relative standard deviation (RSD) was 1.13%; reliability (bias) was 10.37%; the lower limit of quantitation was 0.18 ng/mL; the limit of detection was 0.6 ng/mL. For the measurements in triplicate, the intra-day variability (RSD) was 3.21% and the intervariability was 4.77%.

Plasma AAs were expressed as absolute values and as a percentage (%) of total amino acids (TAAs). We reasoned that AA/TAAs might reflect the body utilization of the specific AA more than changes in absolute values.

#### 2.2.2. Oxidative Stress Markers

8-hydroxy-2’-deoxyguanosine (8-OHdG) and malondialdehyde (MDA) were determined as oxidative stress markers. The measurements were carried out in triplicate in the same laboratory. The mean of the three measurements was calculated and adopted. Their concentration was assessed using a microplate spectrophotometer (BioTek ELx800).

(a) 8-hydroxy-2’-deoxyguanosine. 8-OHdG is a ubiquitous marker of oxidative stress: it is a product of oxidatively damaged DNA formed by hydroxy radical, singlet oxygen and direct photodynamic action [24,25].
  Plasma levels of 8-OHdG were measured using the “Highly Sensitive 8-OHdG Check ELISA kit” (Japan Institute for the Control of Aging 710-1 Haruoka, Fukuroi City, Shizuoka Pref., 437-0122 Japan) according to the manufacturer’s instructions (normal value: 0.1–0.3 ng/mL). To assay properly, pre-treatment of plasma samples is needed for 8-OHdG detection: 8-OHdG ELISA kits may be affected by high-molecular weight substances (>10 kDa) such as protein. To separate these interfering substances, filtration of plasma using an ultrafilter (cut off molecular weight 10 kDa) is necessary (performed with “Microcon-10 kDa Centrifugal Filter Unit”; Merck Millipore, Darmstadt, Germany).
(b) Malondialdehyde. MDA is a naturally occurring product of lipid peroxidation, a biomarker of oxidative stress; therefore, MDA is used as an indicator of oxidative stress. High plasma MDA levels indicate elevated oxidative stress, generally regarded as a pathological condition [26].
  Plasma levels of MDA were measured using the “Cayman’s TBARS Assay Kit” (Cayman Chemical Company, Ann Arbor, MI, USA) according to the manufacturer’s instructions (normal value: 1.86–3.94 µM).

### 2.3. Statistical Analysis

Central tendency and dispersion of collected data were reported as mean ± SD. Within-group comparisons (patients at different observation times) were carried out by repeated measures analysis of variance. 

The association between variables was assessed by correlation analysis (Pearson r). 

A *p*-value < 0.05 was considered to be statistically significant. When appropriate, the false discovery rate (FDR) was controlled at 5% using the Benjamini–Hochberg method and FDR adjusted *p*-values were reported. All analyses were carried out using the SAS/STAT statistical package, release 9.4 (SAS Institute Inc., Cary, NC, USA).

## 3. Results

### 3.1. PAL and MOS during XELOX Therapy

The study showed that throughout the XELOX therapy cycle (from phase A to phase D) PAL and MOS showed no significant changes compared to the values observed in the pre-XELOX therapy phase (A) (Table 3). Only the branched chain amino acid/essential amino acid ratio (BCAAs/EAAs) (Table 3), BCAAs expressed as a % of TAAs and arginine expressed as a % of TAAs (Table 4) were shown to be significantly lower than in the pre-XELOX therapy phase (*p* = 0.017, *p* = 0.028, *p* = 0.028, respectively).

Table 5 showed that plasma levels of MDA were higher than the reference values both in phase A and during XELOX therapy. Moreover, plasma levels of MDA did not change during chemotherapy. 8-OHdG levels were within normal values at all the time points considered (Table 5). 

MDA (Table 5) showed a clear downward trend from phase A to phase D. These changes were statistically significant before correction for multiple comparisons, but significance was lost after FDR correction.

The results showed that the varied dose of the chemotherapeutic drug that patients were actually given (<80% and, respectively, ≥80% of the scheduled dose) did not influence the time courses of PAL and MOS. The reductions in anticancer doses were necessary because of side effects that developed such as neurotoxicity (*n* = 9), neutropenia (*n* = 1), reduced platelet count (*n* = 4) and increased serum creatinine (*n* = 1).

Chemotherapy (Table 2) was associated with increased mean corpuscular volume (MCV), mean content of hemoglobin (MCH) and liver enzyme γ-glutamyl transpeptidase, and with reduced total white cell counts, neutrophil counts and platelet counts. Lymphocyte and monocyte counts, as a % of total white cells, increased over the course of chemotherapy. 

At the initiation of XELOX therapy, the CRC patients had normal body weight, mild anemia and hypoalbuminemia (Table 2). All the patients declared that they resumed their usual diet after surgery. Moreover, the patients declared that they did not reduce their diet except for the first day or two after oxaliplatin infusion because of nausea, some vomiting episodes and gastric fullness. 

### 3.2. Correlations Between Circulating AAs and Markers of Oxidative Stress

The study found that some AAs significantly correlated with MDA in the pre-chemotherapy period (Table 6). During this period, after considering only significant moderate correlations (*r* = 0.3–0.5) [27], aspartic acid, cystine, valine and methionine were negatively correlated with MDA. Histidine, on the contrary, was positively correlated with MDA. No significant correlations were found in the first month (from A to B) of chemotherapy, but an important negative correlation was found between TAAs and MDA (*p* = 0.020) from the first month to the end of XELOX therapy (from B to D) (Table 7). 

## 4. Discussion

The present investigation shows that during XELOX therapy, plasma levels of AAs (PAL) and markers of oxidative stress (MOS) did not change significantly. Only the plasma ratios of BCAA/EAA, BCAAs as a % of TAAs and arginine as a % of TAAs significantly decreased. 

In addition, the study shows the existence of important correlations between plasma levels of amino acids and oxidative stress.

### 4.1. Plasma Amino Acids and Markers of Oxidative Stress during the XELOX Therapy Cycle

The study therefore disproved our hypothesis that XELOX therapy may be associated with the worsening of PAL and oxidative stress. The absence of a negative impact on both PAL and MOS is validated by the fact that the changes in the two parameters were similar between patients who actually received varied XELOX doses (<80% and, respectively, ≥80% of the scheduled doses).

This study suggests that patients had balanced protein intakes/body proteolysis. This may explain the maintenance of PAL during XELOX therapy. BCAAs were probably used for energy production rather than for protein synthesis processes. If BCAAs had mainly been used for protein synthesis, we would have found a balanced BCAA/EAA ratio and not a progressive reduction of the ratio.

It is not possible to exclude that absent/reduced protein synthesis activity of the cancer cells following an eradicated tumor could contribute to maintaining plasma concentrations of AAs, in particular tryptophan, histidine, phenylalanine and isoleucine. First, in cancer environments, tryptophan consumption is high as it is catabolized in immune or inflammatory sites [28]. The availability of tryptophan plays a key role for an adequate cancer cell immune response leading to reduced cancer immune tolerance [28]. Indeed, tryptophan depletion suppresses T cell responses to cancer specific antigens. The availability of tryptophan (and other AAs) might be particularly important when immunotherapy against cancer is used. The success of blocking checkpoint molecules could be counterbalanced/limited by an overall reduction in the immune response of cancer cells. Tryptophan depletion, in tumor environments, causes tumor growth by inducing the suppression of T cell responses to cancer antigens [28]. Second, in cancer cells as well as in healthy cells, isoleucine (and valine) enters the Krebs cycle at the level of succinate for energy production. It is noteworthy that in cervical cancer patients the levels of plasma valine and tryptophan have been shown to increase in the responders to neoadjuvant chemotherapy [28]. In these cervical cancer patients, the plasma concentrations of isoleucine and valine decreased from patients with a complete disappearance of lesions to patients with a stable disease [28]. Third, phenylalanine (together with leucine and tyrosine) is an important amino acid for cell autophagy [29]. This suggests the maintenance of body cell autophagy processes in the study patients. Fourth, histidine is a major component of the heme group; therefore, the maintenance of histidine levels is important for the synthesis of hemoglobin and mitochondrial cytochromes. This may suggest increased mitochondrial damage.

At the initiation of XELOX therapy, the patients were in a state of oxidative stress characterized by peroxidation of cell lipid structures but not by DNA damage. Even though the marker of DNA oxidation was normal, other types of DNA damage, which were not measured in the current study, cannot be excluded. On the other hand, in the patients, pro-oxidant factors were still active, as indicated by high levels of MDA. We believed that, in a context of persisting oxidative stress, the use of therapy strategy inhibiting DNA repair may be important [30,31,32,33,34], especially against cancer cells with a low rate of proliferation, which are poor responders to standard chemotherapy.

In this study, it was not possible to understand the role that pre-surgery cancer plays in determining oxidative stress [35] and whether it increases or decreases after surgical procedures. 

The extent of oxidative stress is likely to be higher than the amount that is documented here by MDA. In fact, we did not measure the reactive nitrogen species (RNS), which, along with reactive oxygen species (ROS), are continuously produced in the muscles of healthy individuals [36]. ROS and RNS modulate muscle contractile function and interact with each other [37]. 

A low arginine/TAA ratio, albeit indirectly, may indicate increased muscle RNS production given that arginine is abundant in skeletal muscle [36] and is the substrate forming nitric oxide (NO) by nitric oxide synthase (NOS).

An important factor contributing to high muscle oxidative stress was the inflammation primed by surgery [38]. After surgery, inflammation causes the muscles to be invaded by phagocytes (neutrophils and monocytes–macrophages), which release ROS, thus damaging muscle cells [37]. 

In the study, the normal plasma concentration of 8-OHdG, a marker of oxidative damage to DNA, was surprising given that ROS might cause mitochondrial dysfunction via mitochondrial DNA damage [39], and even physiological factors such as physical labor, smoking, low meat intake and low BMI significantly increased 8-OHdG levels [40]. We postulated that the normal 8-OHdG in the study patients might therefore be due to the repair of a possible excess of the molecule by 8-oxoguanine DNA glycosylase 1 largely expressed in human cells [39]. Otherwise, oxaliplatin would exert its antitumor efficacy, without causing oxidative DNA damage [41].

Increased oxidative stress is detrimental for muscle mass and for functional capacity [16,42,43,44].

The results of this study were in contrast with recent experimental studies reporting oxaliplatin-induced increased reactive oxygen species formation and skeletal myopathy in free-cancer mice [16]. In that experiment, oxaliplatin administration reduced lean tissue mass that was not associated with nutrition and/or energy expenditure and increased mitochondrial superoxide [16], but with upregulation of the myopathy-linked genes Foxo3, MAFbx and Bnip3 [17].

To explain the discrepancy between the present investigation and the findings of the above studies, apart from the differences that exist between the intact animal models and studies on humans with cancer, we hypothesized that the patients had adequate nutrition/protein intakes. The following factors indicate the patient nutrition adequacy during XELOX therapy. First, the patients maintained their body weight and serum albumin did not worsen. Second, the patients did not complain of serious digestive symptoms such as fullness, nausea, vomiting or diarrhea impacting on appetite and intestinal function. Third, the fact that there was maintenance of circulating AAs at the end of XELOX therapy means that there was a maintenance of an adequate protein intake and/or reduced body (muscle) proteolysis. Theoretically, a disappearance of possible cancer-induced proteolytic agents including HMGB1 [45] could reduce muscle proteolysis. The normal plasma glutamine levels may support the hypothesis [45]. The resumption of patients’ normal eating habits was not monitored in this study. Both this factor and the absence of PAL measurements in the surgical setting make it impossible for us to understand the influence of protein intake on PAL in the time interval between surgery and the initiation of chemotherapy. It is highly likely that the resumption of normal eating habits limited the plasma deterioration of AAs since dietary protein-derived AAs and body/muscle proteolysis [46,47] are normally the determinants of PAL.

The positive effect of adequate nutrition may have contributed to the fact that oxidative stress did not worsen. Indeed, diet protein-derived AAs may preserve mitochondrial function whose alteration increases free radical production. This is supported by two of our study findings, the negative correlation between some circulating AAs and MDA in the pre-chemotherapy phase, and the negative correlation observed between circulating TAAs and MDA from the first month (B) to the end of XELOX therapy (D). The absence of correlations between AAs and MDA during the first XELOX cycle might be attributed to small insignificant changes in the two variables. In addition, adequate nutrition provided patients with antioxidant substances that were able to limit/counteract the excess of free radical production. 

### 4.2. Correlations Between PAL and MOS

The study highlighted the links between plasma AAs and MDA. Indeed, MDA was negatively associated with both plasma AA precursors of glutathione such as cystine, methionine, glycine and important intermediates of TCA cycle activity for energy production such as aspartic acid and valine. Although correlation does not necessarily indicate a cause–effect relationship, we believed the correlations in this study might not just be random, given how essential the above AAs were for both cell glutathione synthesis and mitochondrial energy production. In support of this consideration, supplementation of an EAA mixture has been shown to increase erythrocyte glutathione in subjects with Parkinson’s disease [48].

On the contrary, a strong positive correlation was observed between MDA and histidine. This is surprising, at least to us, because we expected a negative correlation as histidine is an efficient scavenger of ROS [49]. To explain this discrepancy, the plasma levels of histidine may reflect its muscle release in a higher amount than its utilization as a cell scavenger. 

Chemotherapy was associated with increased mean corpuscular volume (MCV), mean content of hemoglobin (MCH) and serum levels of γ-glutamyl transpeptidase, and with reduced total white cell counts, neutrophil counts and platelet counts. Lymphocyte and monocyte counts, as a % of total white cells, increased over the course of chemotherapy. 

We hypothesized that these results were due to two factors: (1) the resumption of normal eating habits (mean hemoglobin content, lymphocyte and monocyte counts), and (2) the toxic effects of chemotherapy (reduced total white cell counts, mean corpuscular volume, neutrophil counts and platelet counts). It is likely that the γ-glutamyl transpeptidase increases were due to oxaliplatin-induced liver injury leading to increased levels of enzymes of cholestasis [50]. 

The study shows that the patients suffered from typical XELOX neurotoxicity [51,52].

### 4.3. Potential Advantages for Patients of Maintenance of Plasma AAs during and after Chemotherapy

There are several potential advantages for patients that result from maintenance of AAs during and after therapy, including:

- Maintenance of overall anabolic activity and body composition, especially in muscle tissue, mainly in subjects with muscle depletion and physical deconditioning. These aspects reduce the risk of developing sarcopenia/cachexia;
- Maintenance of the proliferation and function of immune cells;
- Acceleration of wound healing processes;
- Limitation of the cellular formation of free radicals;
- Increased pain threshold due to a reduction in patients’ perception of pain: this effect is mainly related to the branched chain amino acids (BCAAs) leucine, valine and isoleucine. BCAAs activate the serotoninergic and histaminergic cerebral pathways whose precursors are the plasma AAs tryptophan and histidine, respectively [53].

The maintenance of a normal plasma AA profile over time makes patients more tolerant of possible further therapy, such as chemo/radiotherapy or surgery.

The advantages related to the maintenance of the plasma levels of histidine, threonine, arginine, tryptophan, 3-methyl-histidine and phenylalanine may be particularly interesting. Histidine has numerous biochemical activities that include heme syntheses (mitochondrial cytochromes and hemoglobin) and albumin synthesis [54]. The amino acid threonine is widely used by enterocytes in the small intestine, mainly for the formation of the mucus layer and for immunoglobulin synthesis [55]. A reduction in the plasma concentration of arginine reduces the formation of nitrogen radicals (not measured in our study) [36] in a context where the prooxidant activity of the cells is still active (high levels of MDA were maintained). The maintenance of the plasma concentration of tryptophan can strongly stimulate protein synthesis, including albumin [56,57,58]. Measuring the plasma concentration of 3-methyl-histidine could be clinically informative as it may suggest the level of catabolism of skeletal muscle contractile proteins. A well-planned study is needed to better understand the behavior of 3-methyl-histidine in subjects who have undergone combined surgical/antiblastic therapy. The maintenance of the plasma levels of phenylalanine during chemotherapy plays an important role in inhibiting autophagy proteolysis [29] and in contributing to the body’s anabolic activity as phenylalanine is essential for protein synthesis.

## 5. Limitations and Future Studies

The investigation has several limits, which may be overcome with well-planned studies.

The results should be confirmed in a larger population of patients, including subjects with low body weight [59] at the time of cancer diagnosis and/or post-surgery treatment.

Plasma amino acids and markers of oxidative stress were not measured at the time of cancer diagnosis, before or after the surgical procedure. If the concentrations of both amino acids and markers of oxidative stress in a continuum were known, it may provide better information, albeit indirect, of the evolution over time of body/muscle protein metabolism and free radical species production. 

The study suggests that it is mandatory to monitor nutritional intakes at each step of a patient’s diagnostic-therapeutical (surgery + chemotherapy) itinerary in order to commensurate protein and antioxidant intakes with changes in plasma amino acids and markers of oxidative stress. Monitoring body composition and muscle strength at each step of a patient’s diagnostic-therapeutical itinerary and relating them to plasma amino acids and markers of oxidative stress may provide physicians with more information in order to understand, albeit indirectly, anatomical, biochemical and functional modifications that occur in a patient from the diagnosis of cancer to the end of chemotherapy.

Determining markers of inflammation as part of routine biohumoral variables [38,60,61] can also allow us to better understand muscle/body catabolism and changes in plasma PAL and MOS. 

Measurements of muscle amino acid arteriovenous differences can provide us with information on the net release/uptake of amino acids by muscles.

A well-planned study could document whether normal-weight CRC surgery patients, before initiating chemotherapy, could benefit from a supplemented specific AA mixture targeting muscle protein synthesis and aerobic energy formation. This may be important in the light of subsequent possible chemotherapy-associated side effects such as anorexia and digestive troubles, including mucositis.

## 6. Conclusions

The study shows that in CRC surgery patients, chemotherapy did not affect plasma amino acid levels and plasma markers of oxidative stress. Moreover, the study indicates strict correlations between plasma AAs and markers of oxidative stress.

## 7. Some Useful Information for Nutritional Practice

The results of the current study need to be confirmed in a larger patient population. Having said that, although this research was observational in nature, it might provide some useful information for nutritional practice.

- XELOX therapy did not seem to threaten the metabolism of AAs or to exalt oxidative stress unless patients reduce their nutritional intake. This means that chemotherapy per se did not cause malnutrition.
- Therefore, patients’ nutritional intake, particularly protein, should be monitored. Any loss of appetite and/or digestive troubles arising after surgery or during chemotherapy should be corrected/limited immediately.
- After surgery, patients awaiting chemotherapy should be informed about the importance of a diet with adequate protein-calories and antioxidant substances. Future studies will establish the usefulness of supplemented antioxidant substances.

## Figures and Tables

**Table 1:** Oncologic characteristics of colorectal cancer (CRC) surgery patients.

Patients (*n* = 14)	Differentiation Grades	MAC/TNM Staging Classification [22]
1	G2	C2/pT3pN1a
2	G3	C2/pT4apN2b
3	G2/G3	C2/pT4apN1b
4	G3	C2/pTapN2b
5	G2/G3	C2/pT2pN1b
6	G3	C2/pT4apN2b
7	G3	C2/pT4apN0
8	G3	C3/pT4bpN2b
9	G2	C2/pT2pN2a
10	G2	B2/pT4apN0
11	G2	C2/pT3N1b
12	G2	C2/pT3pN1b
13	G2 and G3 in some areas	B2/pT4apN0
14	G2	C2/pT4pN1A

Abbreviations: MAC, modified Astler-Coller; TNM, tumor, node, metastases. Differentiation grades: G2, moderately differentiated; G3, badly differentiated. B2: lesions penetrating the muscularis propria, with negative nodes.

**Table 2:** Demographic, anthropometric, biohumoral variables of the study patient groups.

Variable	Patients Phase A (*n* = 14)	Patients Phase B (*n* = 14)	Patients Phase C (*n* = 14)	Patients Phase D (*n* = 14)	FDR Adjusted *p*
Age (years)	58.69 ± 9.50	-	-	-	-
Male/female	11/3	-	-	-	-
Body weight (kg)	68.13 ± 11.36	68.33 ± 11.68	69.27 ± 11.84	69.50 ± 10.80	0.17
Body mass index (kg/m^2^)	22.82 ± 3.93	22.87 ± 3.98	23.19 ± 4.07	23.30 ± 4.10	0.17
Albumin (NV 3500–5200 mg/dL)	3042.89 ± 1713.92	2897.37 ± 1563.60	2885.35 ± 1595.51	3200.00 ± 800.00	0.73
Creatinine (NV: M 0.73–1.18 mg/dL; F 0.55–1.02 mg/dL)	0.84 ± 0.25	0.82 ± 0.23	0.83 ± 0.22	0.85 ± 0.21	0.90
Hemoglobin (NV: M 13.2–17.3 g/dL; F 11.7–15.5 g/dL)	12.36 ± 1.51	12.40 ± 1.10	12.76 ± 0.96	12.60 ± 0.94	0.73
Red blood cell count (NV: M 4.30–5.70 × 10^6^/μL; F 3.80–5.20 × 10^6^/μL)	4.38 ± 0.44	4.02 ± 0.32	3.99 ± 0.41	3.85 ± 0.61	0.11
Hematocrit (NV: M 39.0%–49.0%; F 35.0%–45.0%)	37.64 ± 3.65	37.27 ± 2.74	38.54 ± 3.29	37.20 ± 3.10	0.73
Mean corpuscular volume (NV 82.0–98.0 fL)	86.16 ± 7.10	92.90 ± 5.69	97.14 ± 7.48	104.00 ± 8.50	0.007
Mean hemoglobin content (NV 27.0–32.0 pg)	28.27± 2.98	30.89 ± 2.09	32.20 ± 2.87	31.90 ± 2.50	0.009
White blood cell count (NV 4.00–10.00 × 10^3^/μL)	6.50 ± 1.24	4.74 ± 0.94	5.23 ± 1.63	5.70 ± 2.00	0.009
Neutrophil count (NV 2.0–8.0 × 10^3^/μL)	4.26 ± 0.85	2.43 ± 0.60	2.88 ± 1.42	2.75 ± 1.80	0.003
Lymphocyte count (NV 1.5–4.0 × 10^3^/μL)	1.44 ± 0.40	1.47 ± 0.42	1.44 ± 0.49	1.51 ± 0.51	0.97
Monocyte count (NV 0.1–1.0 × 10^3^/μL)	0.57 ± 0.13	0.69 ± 0.15	0.71 ± 0.11	0.68 ± 0.15	0.09
Eosinophil count (NV 0.1–0.5 × 10^3^/μL)	0.23 ± 0.10	0.18 ± 0.04	0.20 ± 0.09	0.22 ± 0.06	0.47
Basophil count (NV 0.0–0.2 × 10^3^/μL)	0.03 ± 0.05	0.03 ± 0.05	0.02 ± 0.04	0.02 ± 0.03	0.90
Platelet count (NV 150–450 × 10^3^/μL)	319.86 ± 161.67	160.86 ± 43.83	147.71 ± 63.93	139.80 ± 66.10	0.018
Total bilirubin (NV 0.20–1.10 mg/dL)	0.43 ± 0.25	0.77 ± 0.45	0.74 ± 0.24	0.77 ± 0.31	0.08
γ-glutamyl transpeptidase (NV 11–53 mU/mL)	39.00 ± 20.99	61.29 ± 57.79	79.43 ± 77.32	81.70 ± 64.91	0.17
Alanine transaminase (NV 11–34 mU/mL)	19.14 ± 9.86	34.71 ± 30.60	23.57 ± 11.12	25.64 ± 10.80	0.20
Aspartate transaminase (NV 11–39 mU/mL)	18.40 ± 11.59	31.40 ± 19.81	29.40 ± 17.53	28.90 ± 15.81	0.18

Data are expressed as mean values ± standard deviation. Statistical analysis: repeated measures analysis of variance. Level of significance: *p* < 0.05. False discovery rate controlled at 5% using the Benjamini–Hochberg method. Abbreviations: FDR, false discovery rate; NV, normal values; M, male; F, female.

**Table 3:** Plasma amino acid concentrations (µmol/L) at each phase of chemotherapy (phase A = before; phase B = 1 month; phase C = 3 months and phase D = at the end of chemotherapy cycle—6 months).

Amino Acids (µmol/L)	Patients Phase A (*n* = 14)	Patients Phase B (*n* = 14)	Patients Phase C (*n* = 14)	Patients Phase D (*n* = 14)	FDR Adjusted *p*
Aspartic acid	8.53 ± 3.34	7.55 ± 3.72	7.65 ± 3.95	12.78 ± 16.31	0.94
Glutamic acid	142.43 ± 28.75	149.85 ± 30.14	142.25 ± 42.79	184.17 ± 144.07	0.94
Asparagine	52.73 ± 19.59	47.69 ± 13.58	51.88 ± 21.85	50.55 ± 24.38	0.94
Serine	43.79 ± 11.52	41.68 ± 13.39	40.78 ± 13.23	49.85 ± 41.59	0.94
Glutamine	292.37 ± 123.43	326.82 ± 159.97	326.04 ± 156.35	245.50 ± 97.45	0.94
Histidine	114.56 ± 37.87	140.30 ± 48.48	136.76 ± 44.46	117.03 ± 48.34	0.94
Glycine	127.95 ± 52.00	148.58 ± 42.82	144.60 ± 45.52	163.99 ± 112.55	0.94
Threonine	84.88 ± 25.60	86.84 ± 25.22	82.90 ± 23.02	83.01 ± 28.20	0.94
Alanine	343.67 ± 95.03	357.68 ± 120.91	360.62 ± 181.10	270.43 ± 87.53	0.94
Arginine	62.83 ± 17.91	58.67 ± 21.86	45.01 ± 16.65	55.75 ± 15.38	0.90
Tyrosine	58.50 ± 15.80	51.34 ± 16.16	59.99 ± 28.36	55.92 ± 15.34	0.94
Tryptophan	52.06 ± 13.23	51.77 ± 16.24	51.86 ± 17.44	55.22 ± 22.02	0.94
Phenylalanine	52.54 ± 11.16	53.31 ± 9.54	60.86 ± 23.55	65.41 ± 31.39	0.94
Isoleucine	63.74 ± 15.03	56.63 ± 18.68	60.54 ± 27.46	60.79 ± 22.63	0.94
Leucine	103.52 ± 28.66	84.32 ± 32.38	87.35 ± 48.30	78.09 ± 34.62	0.94
Lysine	118.78 ± 37.44	126.95 ± 58.45	115.35 ± 65.11	120.83 ± 51.83	0.94
3-methyl-histidine	6.82 ± 5.88	7.49 ± 3.60	4.69 ± 1.95	9.55 ± 10.03	0.94
Valine	193.67 ± 48.69	169.30 ± 58.48	173.26 ± 87.61	241.16 ± 244.45	0.94
Cystine	187.18 ± 72.12	195.89 ± 46.64	189.64 ± 58.93	453.50 ± 866.46	0.94
Methionine	26.96 ± 7.88	26.70 ± 8.41	30.03 ± 12.68	62.15 ± 110.12	0.94
EAAs	696.14 ± 159.57	655.81 ± 197.81	662.15 ± 281.50	766.66 ± 450.02	0.94
BCAAs	360.93 ± 90.40	310.24 ± 107.67	321.15 ± 162.01	380.04 ± 245.80	0.94
BCAAs/EAAs	51.73 ± 3.32	46.68 ± 6.20	47.43 ± 4.41	48.62 ± 3.55	0.017
TAAs	2254.17 ± 492.07	2187.87 ± 578.23	2171.13 ± 723.92	2433.77 ± 1538.81	0.94

Data are expressed as mean values ± standard deviation. Statistical analysis: repeated measures analysis of variance. Level of significance: *p* < 0.05. False discovery rate controlled at 5% using the Benjamini–Hochberg method. Abbreviations: FDR, false discovery rate; EAAs, essential amino acids; BCAAs, branched chain amino acids; TAAs, total amino acids.

**Table 4:** Plasma amino acids expressed as a percentage (%) of total amino acids at each phase of chemotherapy (phase A = before; phase B = 1 month; phase C = 3 months and phase D = at the end of chemotherapy cycle—6 months).

Amino Acids	AA % TAAs Patients - Phase A (*n* = 14)	AA % TAAs Patients - Phase B (*n* = 14)	AA % TAAs Patients - Phase C (*n* = 14)	AA % TAAs Patients - Phase D (*n* = 14)	FDR Adjusted *p*
Aspartic acid	0.41 ± 0.18	0.34 ± 0.17	0.34 ± 0.13	0.44 ± 0.18	0.34
Glutamic acid	6.82 ± 1.42	7.02 ± 1.16	6.80 ± 1.81	7.35 ± 1.61	0.53
Asparagine	2.50 ± 0.90	2.20 ± 0.40	2.41 ± 0.71	2.17 ± 0.47	0.47
Serine	2.06 ± 0.38	1.91 ± 0.33	1.90 ± 0.35	1.94 ± 0.40	0.20
Glutamine	13.40 ± 3.95	14.43 ± 4.59	14.68 ± 4.40	11.93 ± 4.86	0.34
Histidine	5.38 ± 1.47	6.51 ± 1.86	6.56 ± 1.94	5.86 ± 2.46	0.20
Glycine	5.92 ± 1.54	6.87 ± 1.64	6.85 ± 1.99	6.59 ± 1.34	0.20
Threonine	3.96 ± 0.81	4.00 ± 0.91	3.97 ± 0.92	3.95 ± 1.44	1.00
Alanine	16.01 ± 1.96	16.22 ± 2.16	16.08 ± 2.78	13.69 ± 5.02	0.34
Arginine	3.02 ± 0.94	2.69 ± 0.68	2.16 ± 0.86	2.66 ± 1.12	0.028
Tyrosine	2.78 ± 0.65	2.35 ± 0.51	2.70 ± 0.65	2.63 ± 0.89	0.47
Tryptophan	2.47 ± 0.54	2.36 ± 0.37	2.40 ± 0.29	2.42 ± 0.40	1.00
Phenylalanine	2.49 ± 0.36	2.49 ± 0.37	2.80 ± 0.42	2.81 ± 0.47	0.062
Isoleucine	2.99 ± 0.32	2.59 ± 0.58	2.74 ± 0.50	2.69 ± 0.53	0.34
Leucine	4.85 ± 0.80	3.83 ± 1.07	3.89 ± 1.01	3.96 ± 1.70	0.20
Lysine	5.60 ± 1.49	5.75 ± 1.78	5.20 ± 1.67	5.36 ± 1.65	0.47
3-methyl-histidine	0.31 ± 0.29	0.34 ± 0.17	0.22 ± 0.12	0.33 ± 0.14	0.48
Valine	9.13 ± 1.48	7.71 ± 1.76	7.78 ± 1.54	8.98 ± 2.40	0.20
Cystine	8.67 ± 2.09	9.23 ± 2.57	9.17 ± 3.10	12.49 ± 11.20	0.50
Methionine	1.27 ± 0.30	1.22 ± 0.24	1.38 ± 0.27	1.82 ± 1.35	0.34
EAAs	32.76 ± 3.99	29.95 ± 4.49	30.16 ± 3.98	31.99 ± 4.61	0.21
BCAAs	16.97 ± 2.46	14.13 ± 3.28	14.41 ± 2.90	15.63 ± 2.99	0.028

Data are expressed as mean values ± standard deviation. Statistical analysis: repeated measures analysis of variance. Level of significance: *p* < 0.05. False discovery rate controlled at 5% using the Benjamini–Hochberg method. Abbreviations: AA, amino acid; FDR, false discovery rate; EAAs, essential amino acids; BCAAs, branched chain amino acids; TAAs, total amino acids.

**Table 5:** Plasma concentrations of malondialdehyde (MDA; µM) and 8-hydroxy-2’-deoxyguanosine (8-OHdG; ng/mL) within the patient group at each phase of chemotherapy (phase A = before; phase B = 1 month; phase C = 3 months and phase D = at the end of the chemotherapy cycle—6 months).

	Patients Phase A (*n* = 14)	Patients Phase B (*n* = 14)	Patients Phase C (*n* = 14)	Patients Phase D (*n* = 14)	FDR Adjusted *p*
MDA (NV 1.86–3.94 µM)	8.61 ± 3.03	7.68 ± 2.67	7.32 ± 2.40	6.34 ± 1.62	0.21
8-OHdG (NV 0.1–0.3 ng/mL)	0.15 ± 0.03	0.16 ± 0.02	0.14 ± 0.03	0.14 ± 0.01	0.41

Data are expressed as mean values ± standard deviation. Statistical analysis: within-group comparison (patients at different observation times) carried out by repeated measures analysis of variance. Level of significance: *p* < 0.05. False discovery rate (FDR) controlled at 5% using the Benjamini–Hochberg method. Abbreviations: NV, normal values.

**Table 6:** Correlations (Pearson *r*) between plasma amino acids and malondialdehyde (MDA) found in the patient group at phase A.

Amino Acids	vs. MDA	
	*r*	*p*
Aspartic acid	−0.53	0.044
Glutamic acid	−0.47	0.08
Asparagine	−0.23	0.41
Serine	−0.49	0.061
Glutamine	0.07	0.79
Histidine	0.67	0.006
Glycine	−0.33	0.23
Threonine	−0.05	0.86
Alanine	0.46	0.09
Arginine	−0.08	0.78
Tyrosine	0.11	0.69
Tryptophan	−0.15	0.58
Phenylalanine	−0.49	0.064
Isoleucine	−0.40	0.14
Leucine	0.31	0.26
Lysine	−0.31	0.26
3-methyl-histidine	−0.50	0.08
Valine	−0.53	0.041
Cystine	−0.52	0.046
Methionine	−0.52	0.048
EAAs	0.49	0.066
BCAAs	0.26	0.34
NEAAs	0.46	0.08
TAAs	0.18	0.52

Level of significance: *p* < 0.05. Abbreviations: EAAs, essential amino acids; BCAAs, branched chain amino acids; NEAAs, non-essential amino acids; TAAs, total amino acids.

**Table 7:** Correlations (Pearson *r*) between plasma amino acid and malondialdehyde (MDA) found in the patient Group at phase B, phase C and phase D.

Amino acids	vs. MDA	
	*r*	*p*
Aspartic acid	−0.07	0.81
Glutamic acid	−0.01	0.98
Asparagine	−0.03	0.90
Serine	−0.15	0.58
Glutamine	−0.44	0.09
Histidine	−0.14	0.61
Glycine	−0.11	0.68
Threonine	−0.48	0.062
Alanine	−0.30	0.26
Arginine	−0.40	0.13
Tyrosine	−0.42	0.11
Tryptophan	−0.40	0.13
Phenylalanine	−0.21	0.43
Isoleucine	−0.38	0.15
Leucine	−0.28	0.29
Lysine	−0.35	0.18
3-methyl-histidine	−0.16	0.59
Valine	−0.13	0.64
Cystine	−0.02	0.94
Methionine	−0.04	0.89
EAAs	−0.29	0.28
BCAAs	−0.34	0.20
NEAAs	−0.31	0.25
TAAs	−0.58	0.020

Values were obtained by averaging the measurements at phase B, phase C and phase D. Level of significance: *p* < 0.05. Abbreviations: EAAs, essential amino acids; BCAAs, branched chain amino acids; NEAAs, non-essential amino acids; TAAs, total amino acids.
